# A biphasic effect of age on CPAP adherence: a cross-sectional study of 26,343 patients

**DOI:** 10.1186/s12931-023-02543-x

**Published:** 2023-09-27

**Authors:** Arnaud Prigent, Clément Blanloeil, Anne-Laure Serandour, Franck Barlet, Frédéric Gagnadoux, Dany Jaffuel

**Affiliations:** 1 Groupe Médical de Pneumologie, Polyclinique Saint-Laurent, 35000 Rennes, France; 2 Centre du Sommeil Polyclinique Saint Laurent, 35000 Rennes, France; 3 Elia Medical Ouest, Cesson-Sévigné, France; 4 SLB Pharma, Rennes, France; 5 i-GEIA, Boulogne-Billancourt, France; 6 Service de Pneumologie et Médecine du Sommeil, CHU d’Angers, Angers, France; 7 INSERM, CNRS, MITOVASC, Equipe CarME, SFR ICAT, Université d’Angers, Angers, France; 8 Département de Pneumologie, Hôpital Arnaud de Villeneuve, CHRU de Montpellier, Montpellier, France; 9 Inserm U1046-CNRS 9214-Physiologie et Médecine Expérimentale Cœur et Muscle, Université de Montpellier, Montpellier, France

**Keywords:** CPAP, Elderly people, Adherence, AHI, Leaks

## Abstract

**Background:**

As the prevalence of OSA increases in older patients, the proportion of elderly patients treated with CPAP is expected to become even higher. We studied CPAP-adherence in a real-life cohort involving a large population of elderly patients with OSA.

**Methods:**

eQUALISAS is a cross-sectional study of CPAP treated OSA patients for at least 4 months who received remote monitoring during 2021. CPAP adherence, device-reported residual AHI (AHI_CPAP_) and 95th percentile non-intentional leaks were software collected during January 2021. Age and sex were also collected.

**Results:**

Data from 26,343 patients including 1656 patients aged [80–85] years and 639 patients aged ≥ 85 years were analysed. Median CPAP adherence increases from 6.3 h (< 50 years) to 7 h (75–80 years) and decreases after this age (p < 0.001). The decrease in CPAP adherence observed after the age of 80 was associated with an increase in the proportion of patients with a CPAP-adherence < 4 h/day (p < 0.001). Proportion of CPAP treated female, patients having AHI_CPAP_ mean ≥ 10 events per hour or 95th percentile non-intentional leaks mean over 24 l/min increase with aging of the population. However, for patients over 80 years old, Chi square test showed no association of adherence with gender, AHI_CPAP_ and leaks (p > 0.05).

**Conclusion:**

This study demonstrates that adherence gradually increases with age until 80 years. The proportion of non-adherent patients grows with age after 80 years old. No significative relationship was found between adherence after 80 years old and gender, leaks and AHI_CPAP_.

*Trial registration*: The study is registered on Health Data Hub platform (No. F20220715144543).

## Take home message

Despite the fact that most elderly and very elderly CPAP treated patients show a good adherence for about 7 h per day, there’s a growing proportion of non-adherent patients after the age of 80. Neither high leaks, high AHI_CPAP_ nor gender have been related to the non-adherence in the elderly population.

## Background

Obstructive sleep apnoea (OSA) is one of the most frequent chronic diseases, affecting nearly one billion people worldwide [1]. Continuous positive airway pressure (CPAP), the first line therapy for OSA [2] significantly improves symptoms and quality of life if well adhered to [3]. All around the world, individuals over 65 years old constitute the fastest growing part of the population. As the prevalence of OSA increases in older patients, the proportion of elderly or very elderly patients (≥ 80 years) treated with CPAP is expected to become even higher [4]. OSA in elderly adults is a great challenge for the future. Real-life data on CPAP adherence in very elderly are scarce [5]. With the aim to improve our knowledge in this area, we studied CPAP-adherence in a real-life cohort involving a large subpopulation of very elderly patients with OSA.

## Methods

eQUALISAS is a cross-sectional study of CPAP treated OSA patients for at least 4 months who received remote monitoring during 2021. The primary objective of the eQUALISAS study was to investigate the effect of age on CPAP adherence, the secondary objective was to compare the CPAP adherence of patients older than 80 years with those who are younger.

One-month de-identified CPAP-adherence data were assessed in January 2021 from a healthcare provider database (ELIA Medical). Data were collected using the same CPAP-software (Airsense 10, Resmed). CPAP adherence, device-reported residual AHI (AHI_CPAP_) and 95th percentile non-intentional leaks were software collected during January 2021. For each patient, on January 2021, adherence mean was calculated considering the daily data of the telemonitoring. For AHI_CPAP_ and leaks, daily values were considered only if use was above 2 h. If there were less than 14 considered values per month, means of AHI and leaks were not calculated (n = 2241). Good adherence was defined as a monthly CPAP mean ≥ 4 h/night. Age and sex, number of years of use were also collected.

Data use and confidentiality were ensured by following European Regulation no. 2016-679 on the protection of personal data, and the French Data Protection Authority (CNIL) reference methodology MR004 concerning research reusing previously collected data (Deliberation no. 2018-155 of 3 May 2018); the study has been registered on Health Data Hub platform (N° F20220715144543). All patients were provided with written information about the study and were given the possibility to refuse the use of the collected data. All included patients gave their consent for data collection and anonymization.

For statistical analyses, continuous data were presented as medians with their associated interquartile ranges (IQR) due to non-Gaussian distributions. Qualitative parameters were presented as numbers and percentages. Kruskal–Wallis test was used to compare more than two independent medians. If Kruskal–Wallis test was significant, Dunn test was used for non-parametric multiple comparisons with a Bonferroni correction. Mann–Whitney non- parametric tests were used to compare continuous data in two groups. Receiver operating characteristic (ROC) curves were used to determine the cut-off values of AHI_CPAP_ and leaks for predicting adherence < 4 h. P < 0.05 was considered as a statistically significant difference. For patients over 80 years old, chi-square tests were performed to examine the association between each independent variable (leaks, AHI_CPAP_, gender) and CPAP adherence.

## Results

**Fig. 1:**
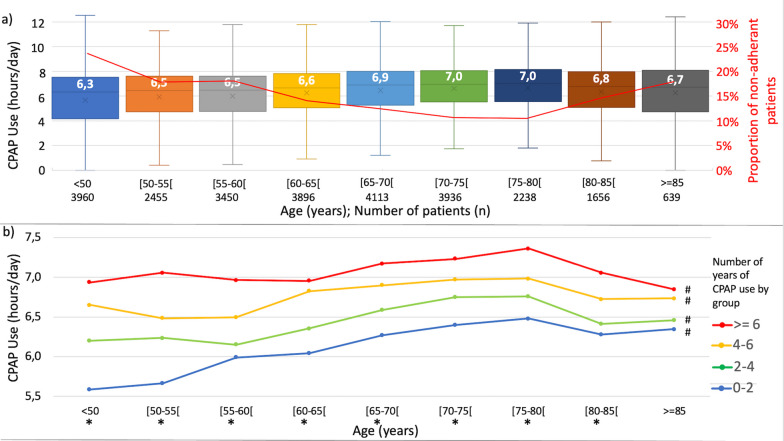
CPAP use (h/day) by age, number of years of use by age. **a** CPAP use by age. Values expressed as median during January 2021. P < 0.001 (Kruskal–wallis test). Red curve: proportion of non-adherent patients (mean adherence < 4h/day) by age group. **b** CPAP use by age group and by number of years of use group. *P < 0.05 (Kruskal–Wallis test) significant difference between number of years of use groups. #P < 0.05 (Kruskal–Wallis test) significant difference between age groups

Data from 26,343 patients (67.7% male, median age 64 years old [interquartile range (IQR) = 17] including 1656 patients aged [80–85] years and 639 patients aged ≥ 85 years) were analysed. The included patients represent 49.2% of the whole population treated by the home care provider during the study period. Median CPAP adherence increases from 6.3 [IQR = 3.3] hours (< 50 years) to 7 h [IQR = 2.6] (75–80 years) and decreases after this age (p < 0.001) (Fig. 1a). Median CPAP adherence remained stable even after 80 years old in adherent patients (mean CPAP use ≥ 4 h/night). Conversely, in the non-adherent group, CPAP adherence increases from 1.6 [IQR = 3.0] hours (< 50 years) to 2.5 [IQR = 2.4] hours (75–80 years) and decreases after this age (p < 0.001). The proportion of non-adherent patients by age group decreases from 24 to 11% at 80 years old and increases after to reach 19% (Fig. 1a). The proportion of patients recently treated (use for 4 months to 2 years and 2 to 4 years) regularly decreases with aging from 32% to 13,1% and from 28% to 16.1%, respectively. As shown in Fig. 1b, the higher the number of years of use is, the higher PAP adherence is (p < 0.05) (except for age group above 85 (p > 0.05)). PAP adherence significantly changes according to each year group use (p < 0.05).

**Fig. 2:**
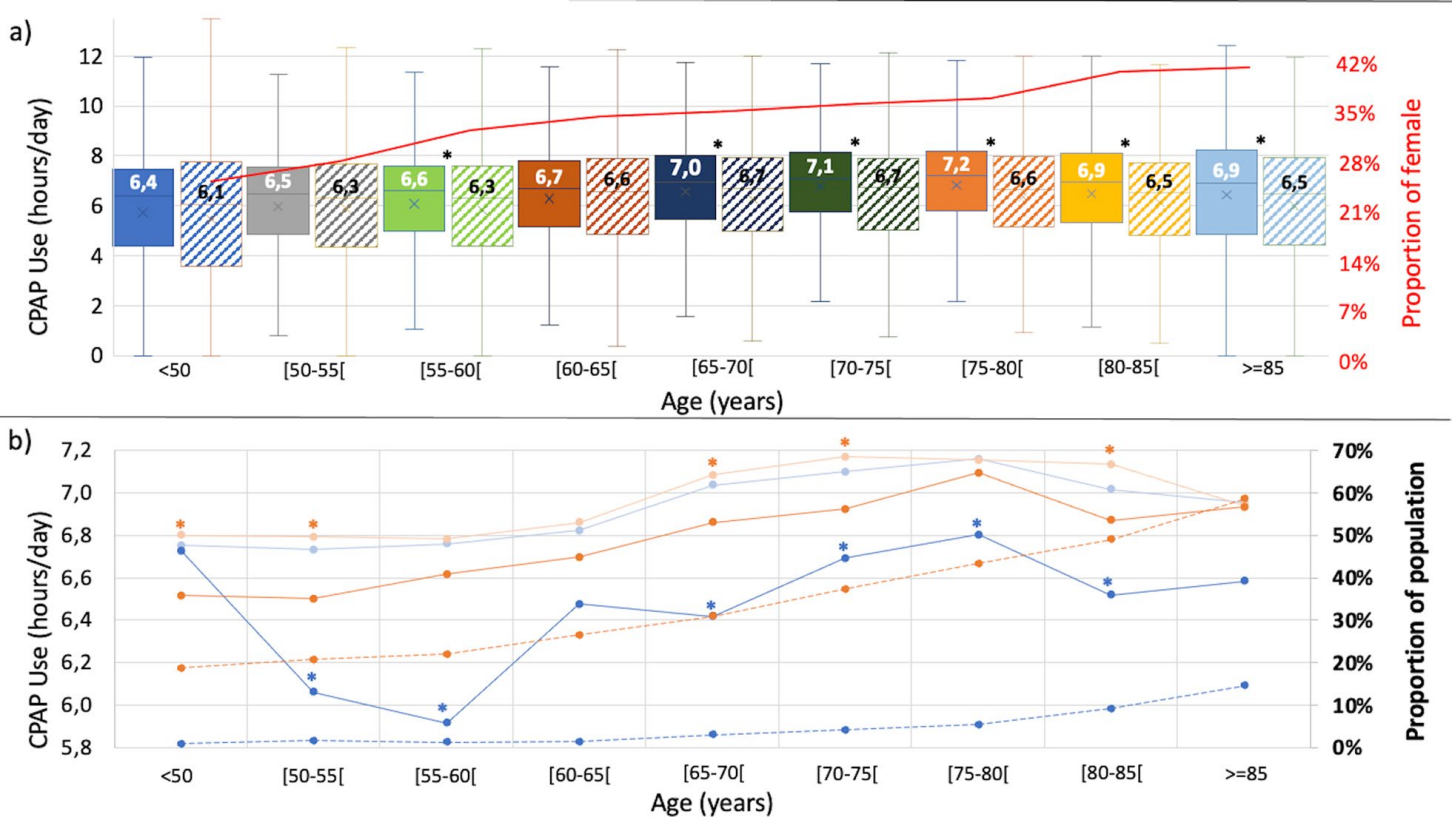
CPAP use (h/day) by age, AHI_CPAP_ and 95th percentile non-intentional leaks. **a** CPAP use by age and by gender (female: striped boxes). Values expressed as median during January 2021 (significant difference between age group for male and female P < 0.001 (Kruskal–Wallis test). *p value < 0.05 (Mann-Withney test): significant difference of adherence between female and male for each age group. Red curve: proportion of female by age group. **b** median CPAP adherence during January 2021 by age by groups of 95th percentile non-intentional leaks over 24 l/min (orange curve), 95th percentile non-intentional leaks under 24 l/min (light orange curve), AHI_CPAP_ ≥ 10 events per hour (blue curve:), AHI_CPAP_ < 10 events per hour (light blue curve:). *p value < 0.05 (Mann–Withney test): adherence between groups AHI and leaks by age groupe. Proportion of population having monthly mean of 95th percentile non-intentional leaks over 24 l/min (orange dashed line) and monthly mean of AHI_CPAP_ ≥ 10 events per hour (blue dashed line) in the general population

The proportion of female increased with the aging of the population (Fig. 2a). CPAP adherence was constantly higher in male compared to female patients whatever the age-group (For details see Fig. 2a). Regardless gender, median adherence follows the same trend than the general population. Patients having a monthly mean of AHI_CPAP_ ≥ 10 events per hour or of 95th percentile non-intentional leaks over 24 l/min used significantly less their CPAP (p < 0.05; for details see Fig. 2b). The proportion of patients having AHI_CPAP_ mean ≥ 10 events per hour or 95th percentile non-intentional leaks mean over 24 l/min in each age group, increases with age (Fig. 2b). The area under the receiver operating characteristic curve for a threshold of adherence < 4 h for AHI_CPAP_ and leaks was respectively of 0,491 (p value = 0.205) and 0.485 (p value = 0.032). For patients over 80 years old, Chi square test shows no dependence between adherence and gender, AHI_CPAP_ and leaks (p > 0.05).

## Discussion

To the best of our knowledge, the eQUALISAS study is the largest real-life study reporting CPAP adherence in very elderly patients treated for at least 4 months. Our study showed: (i) an increase in CPAP adherence up to the age of 80 years and then a slight decrease; (ii) the decrease in CPAP adherence over 80 years old was associated with an increase in the proportion of patients with a CPAP-adherence < 4 h/day; (iii) Increasing age was associated with a higher proportion of females with aging of the population (iv) The proportion of patients having AHI_CPAP_ mean ≥ 10 events per hour or 95th percentile non-intentional leaks mean over 24 l/min increased with age.

It has been shown that PAP adherence increases with the number of years of use [6]. Our study found the same significant association (except for age group above 85 years old). Younger age group have greater proportion of patients recently CPAP treated, participating in the trend that we observed in the general population. However, our study confirms that age is indeed an influencing factor on adherence to CPAP therapy: for the same level of number of years of use in treatment (especially for a use of less than 6 years), median adherence increases between under 50 years old to 80 years old and decreases after this age.

In a previous study including only 262 elderly patients (aged between 76.7 and 87.7 years) newly diagnosed with OSA, the mean adherence time in adherent patients was 6.9 h versus 2 h in non-adherent patients and 85.5% were adherent [7]. Patel and al. described the same evolution regarding low adherers (< 1h) [8] in population over 80 with no precision about the number of patients.

In our opinion, the linearly decrease in total sleep time with age (about 10 min per decade) couldn’t explain this trend [9]. Therefore, the relationship between the decrease of the adherence and gender, leaks and AHI_CPAP_ was explored. Concerning gender, as observed in previous studies [8, 10], we report that females have lower adherence than men, regardless of age. Moreover, proportion of females constantly increases with age. Despite this, after 80 years old, gender and CPAP adherence are not associated (Chi square test > 0.05). Concerning residual leaks and AHI_CPAP_, as shown before in a younger CPAP treated population [10], a constant age-related increase of non-intentional leaks and AHI_CPAP_ was observed in the present study. CPAP adherence was significantly lower in patients having AHI_CPAP_ mean > 10 events per hour or 95^th^ percentile non-intentional leaks mean over 24 l/min in most of age groups but clinically significant (> 30 min/day) for only age groups 50–60 and 65–70 years old with AHI_CPAP_ > 10 events per hour. On the other hand, non-intentional leaks and residual AHI_CPAP_ were not associated with CPAP adherence after 80 years old suggesting that the higher proportion of non-adherers patients after this age was not related to these factors. Finally, no significative association was found between adherence after 80 years old and gender, leaks and AHI_CPAP_.

As an alternative hypothesis, we suggest that the decrease in CPAP adherence after 80 years might be explained by numerous factors including the age-related increase in wake after sleep onset (WASO) [9]. It is obvious that an early morning wake up is an opportunity to stop CPAP prematurely. Arousals and associated movements generate mask displacement and could also explain the higher rate of leaks in this population of patients. Other factors contributing to the decrease in CPAP adherence after 80 years old included chronic sleep problems [11], loneliness (widowed women) [12], less social support, changes in sensory perception/loss of autonomy that may make CPAP use more difficult, different attitudes and beliefs about OSA and CPAP than younger adults. Prevalence of heart failure and stroke [13] increases with age and is associated with poorer sleep quality (but also higher residual AHI_CPAP_).

## Limits and strengths of the study

Our population was treated in France on a long-term basis and our conclusions may not be applicable to short-term and/or other countries. Because of the study design and data collection, we were unable to report more phenotypic data, and obviously important variables (such as comorbidities, residual sleepiness, and partner presence) were unavailable.

Reporting CPAP adherence in a very large population of old and very old people constitutes the strength of this study.

## Conclusions

In conclusion, this study demonstrates that, in a population treated by CPAP for at least 4 months, adherence gradually increases with age until 80 years. The proportion of non-adherent patients increases with age after 80 years old, despite the lower proportion in this age group of patients recently CPAP treated. Strategies including mask selection (easier to fit), and CPAP telemonitoring (alert thresholds adapted to the age) need to be developed in old patients.

## Data Availability

The datasets used and/or analyzed during the current study are available from the corresponding author on reasonable request.

## Abbreviations

AHI: Apnoea-hypopnea index
OSA: Obstructive sleep apnea
IQR: Interquartile range
